# An fMRI Study of Grammatical Morpheme Processing Associated with Nouns and Verbs in Chinese

**DOI:** 10.1371/journal.pone.0074952

**Published:** 2013-10-11

**Authors:** Xi Yu, Yanchao Bi, Zaizhu Han, Sam-Po Law

**Affiliations:** 1 Division of Speech and Hearing Sciences, the University of Hong Kong, Hong Kong SAR; 2 National Key Laboratory of Cognitive Neuroscience and Learning, Beijing Normal University, Beijing, China; Stony Brook University, United States of America

## Abstract

This study examined whether the degree of complexity of a grammatical component in a language would impact on its representation in the brain through identifying the neural correlates of grammatical morpheme processing associated with nouns and verbs in Chinese. In particular, the processing of Chinese nominal classifiers and verbal aspect markers were investigated in a sentence completion task and a grammaticality judgment task to look for converging evidence. The Chinese language constitutes a special case because it has no inflectional morphology per se and a larger classifier than aspect marker inventory, contrary to the pattern of greater verbal than nominal paradigmatic complexity in most European languages. The functional imaging results showed BA47 and left supplementary motor area and superior medial frontal gyrus more strongly activated for classifier processing, and the left posterior middle temporal gyrus more responsive to aspect marker processing. We attributed the activation in the left prefrontal cortex to greater processing complexity during classifier selection, analogous to the accounts put forth for European languages, and the left posterior middle temporal gyrus to more demanding verb semantic processing. The overall findings significantly contribute to cross-linguistic observations of neural substrates underlying processing of grammatical morphemes from an analytic and a classifier language, and thereby deepen our understanding of neurobiology of human language.

## Introduction

Languages vary widely in the complexity of their morphosyntactic system. For instance, on one end of the spectrum, the Chinese language is well-known for its impoverished inflectional morphology [1], [2]. The morphological and phonological structures of Chinese words stay the same during sentence construction. There is only aspectual marking for verbs, and no inflectional marker for nouns in the traditional sense. On the other end of the continuum, there are languages with rich inflectional morphology such as Italian, Polish, Hungarian, where the verb may change its form on the basis of tense, aspect, person, finiteness, negation, or modality, and a noun may be marked for gender, number, or case. Moreover, nouns and verbs in such languages may have different declensional and conjugational patterns, respectively, depending on their classification. Understanding whether and how representation of morphosyntactic processing at the brain level may differ as a function of complexity will significantly inform us about neurobiology of language [3], and possibly contribute to cross-linguistic studies of first language acquisition [4], second language acquisition [5], and bilingualism or multilingualism.

### Neural Substrates of Morphosyntactic Processing in European Languages

Early evidence for neural representation of a morphosyntactic component in the language system comes from behavioral observations of individuals with brain injury. A case study of an Italian-speaking individual with aphasia described a pattern of selective impairment to inflectional morphemes in spontaneous production of sentences and repetition of single words, including nouns, verbs, and adjectives, and relatively preserved production of derivational morphemes [6]. Specific disruption to inflectional morphology was similarly reported of an English aphasic speaker in a reading aloud task [7]. Subsequent case studies show that impaired production of inflectional morphology may occur to specific grammatical class, for instance, to verbs in English [8] and Greek [9] or nouns [10]. That homonyms (to watch, a watch) and pseudowords were used as stimuli for the noun and verb conditions in [8], [10] reduced the possibility that the dissociation patterns were confounded with psycholinguistic factors, and thereby demonstrated that inflectional morphology is specified for grammatical word class in the brain. These findings also suggest distinctive neural correlates of verbal and nominal inflectional morphology.

In the past decade, a number of functional imaging studies employing tasks that explicitly involved operations of inflectional morphology associated with nouns and verbs were conducted to identify brain areas of noun-specific and verb-specific morphosyntax (see [3] for a review of studies showing a left-lateralized fronto-temporal network supporting the processing of inflected spoken words). In most of these investigations, the participants were asked to provide singular-plural alternations for nouns and present-past tense alternations or person agreement for verbs in phrasal or sentential contexts. In a series of studies [11], [12], [13], real words including abstract and concrete nouns and verbs with regular and irregular inflections, as well as pseudowords served as stimuli. Pseudowords were used in the attempt to eliminate semantic confounds between nouns and verbs. Greater activation was found in the left middle frontal gyrus (LMidFG) and bilateral superior posterior parietal regions for verb production and the left middle fusiform gyrus for noun production across conditions of lexicality, concreteness, and regularity of inflection in English [11]. Different cortical regions for production of nominal and verbal inflections were also reported in German, albeit in somewhat different areas – the left superior frontal gyrus extending anteriorly for verbs and the right superior temporal gyrus and the left fusiform gyrus for nouns – in a PET study [12].

The role of LMidFG, particularly its anterior portion (LaMidFG), in the processing of verbal inflection in English was highlighted in [13] using transcranial magnetic stimulation (TMS). Application of repetitive TMS (rTMS) to LaMidFG significantly slowed down morphological operation of verbs but not nouns. This observation was replicated in [14]. Moreover, the study found that production of both regularly and irregularly inflected verbs was inhibited, and the suppression resulting from application of rTMS was restricted to LaMidFG since no word class specific interference was noted in the neighboring Broca's area or the posterior MidFG, or the right homologue of aMidFG. More recently, a linear decrease in activation was reported in the junction of the left inferior frontal gyrus (LIFG) and LMidFG to English inflected verb production over the course of a morphological transformation task [15]. The functional magnetic resonance (fMR) adaptation of the area was argued to be associated with repeated processing of verbal inflections. The LMidFG (Brodmann area (BA)9 and BA10) and LIFG (BA44 and BA45), in addition to the left inferior precentral gyrus (BA6), have similarly been reported to selectively activate for inflected verbs in Italian compared with repetition of verbs as a baseline [16]. In short, there is evidence suggesting that LMidFG underlies morphosyntactic processing of verbs.

However, the claim that an area in the left frontal region can be identified for processing of inflectional morphology specific to verbs has been challenged. More specifically, the left BA44/45 and BA47 were found to be more strongly activated for inflected nouns than verbs in English [17]. The researchers attributed the observation to greater processing difficulty of the noun stimuli, due to lower frequency and irregular inflections, compared to the verb stimuli. It has also been explicitly argued that the activity of the left frontal region is modulated by processing demands instead of reflecting operations of any particular grammatical class [18]. Differing from the studies reviewed thus far, Italian-speaking participants in [18] named pictures depicting events using infinitive verbs, inflected verbs, and action nouns. Since action nouns are not the preferred responses to pictured actions, learned later in life, and morphologically derived from inflected verbs and therefore most complex among the three response types, it was predicted that production of action nouns would elicit the strongest activation. The results confirmed the prediction. BA44 and BA45/47 were significantly more activated for action nouns than uninflected and inflected verbs, while the last two conditions did not differ. It was concluded that the so-called grammatical class effects in the left frontal region were the results of a difference in morphological complexity and/or selection demands between word classes.

The view that the LIFG reflects computational demands from selection among phonological (e.g. [19]) or semantic (e.g. [20], [21]) competitors is quite widely adopted; however, it does not necessarily rule out the possibility that some other area in the left frontal region supports morphological processes specific to a grammatical class. In fact, it is not easy to explain the observation of the recruitment of LaMidFG in production of verbal inflections in English on a selection demands account alone. The past-present tense or third person singular-plural alternations for verbs are not more complex than the singular-plural transformation for nouns in terms of combinatorial pattern or number of alternative responses; furthermore, the singular-plural alternations (of third person) for verbs and nouns have the same phonological form. In other words, there is no intrinsic inconsistency between the results of [18] and those of [11], [13], [14], [15]. While stronger activation for inflectional operations specific to verbs may be driven by variables such as processing complexity, the effects in LaMidFG are still best explained by reference to verb inflection per se.

### The Present Study

This study investigated the neural substrates of grammatical morpheme operations associated with nouns and verbs in Mandarin Chinese. Chinese stands in stark contrast with those languages that have been examined with neuroimaging methods, most notably in terms of inflectional morphology. As mentioned earlier, the Chinese verb is only marked for aspect, and nouns are not marked for number, case, or gender. As such, the claim that word classes, such as nouns and verbs, are not distinctive categories in the Chinese grammar has been made, and it has apparently been supported by null findings of separate neural correlates of nouns and verbs from the lexical decision task [22], [23], [24]. However, word class effects from semantic tasks have recently been reported. Converging evidence from a semantic relatedness judgment task and a semantic associate production task revealed a task-independent region in the left posterior superior and middle temporal cortices (LpSTG&MTG) that activated more strongly for verbs than nouns [25], [26]. Therefore, the proposition that impoverished inflectional morphology would lead to a lack of word class distinction in a language needs to be reconsidered.

While Chinese lacks inflectional morphology, there are grammatical morphemes that take part in the syntax of the language typical of an analytic language, including those relevant to nouns and verbs. They appear before or after the content word without changing its form. There are five aspect markers in Mandarin Chinese [27], the perfective *le5*, experiential *guo4*, and continuous *zhe5*, which may be attached to the end of a verb, the progressive *zai4*, which occurs before a verb, and the delimitative *yi1*, which appears between a verb and its reduplicated form, i.e. V-*yi1*-V.

As for nouns, there is a class of morphemes called classifiers that must appear when a noun is preceded by a numeral and/or a demonstrative, such as *yi1 ben3 shu1* ‘one + classifier + book’. In other words, Chinese is also a classifier language. Classifier languages are spoken by a large portion of the world's population, including speakers of East and Southeast Asian languages, some Australian aboriginal languages, and some native American languages [28]. Two major types of classifiers can be distinguished in Chinese, sortal (or count-classifiers) and mensural (or mass-classifiers). The former are closed-class morphemes and often related to the noun, especially when it denotes an object, in terms of shape, animacy, function or social status [28], [29], [30], while the latter are open-class morphemes that quantify the amount of an object or objects (e.g. a *group* of students, a *glass* of water, a *month* of work). Estimates of the number of classifiers vary widely across sources, ranging from two dozen to several hundreds depending on whether mensural classifiers are also included. In Mandarin, it has been estimated that there are over 60 classifiers [1], but only about two dozen are “core classifiers” for most classifier use. This is consistent with the description in [28], which listed 126 “classifiers” but indicated that only 19 functioned solely as classifiers.

Given the relationship between nouns and sortal classifiers, it is not surprising that sortal classifiers have been studied extensively in child language development (e.g. [31], [32]) and on the relationship between language and cognition (e.g. [28], [33], [34]). Nonetheless, it should be emphasized that there is “a non-trivial degree of arbitrariness” (p. 1127 in [28], and [34]) in the choice of a sortal classifier from the meaning of the noun (e.g. the classifier *pi3* for both ‘horse’ and ‘bolts of cloth’). Moreover, not only concrete nouns or objects (which have been the focus of most previous work on classifiers) but also abstract nouns require a classifier (e.g. ‘news’, ‘hope’, ‘resentment’), and it is hard to discern any physical or functional relationship between the abstract noun and its classifier. It is also worth mentioning that in casual speech, most Mandarin Chinese speakers would use the general classifier 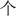 *ge4* in place of the proper classifier. Given all these characteristics, it has been proposed that classifiers can be seen as the Chinese counterparts of noun inflection [27], [35].

The existence of sortal classifiers and aspect markers associated with Chinese nouns and verbs constitutes a very special case from the perspective of cross-linguistic study. The relative simplicity of the Chinese system raises the question of whether the degree of complexity or richness of a grammatical component would affect its representation in the brain, analogous to previous reports of null findings for representation of lexical (or derivational) morphology in English (e.g. [36], [37] but see [38]), but positive findings in Hebrew (e.g. [39]), German (e.g. [40]), and Italian (e.g. [16], [41]). In addition, contrary to most European languages in which the verbal paradigm is more complex than the nominal counterpart, the contrast between the nominal classifier and verbal aspect marker inventories in Chinese presents the opposite pattern. This difference renders Chinese a highly interesting testing ground for assessing the view that neural correlates of morphosyntactic processes, particularly in LIFG, specific to a grammatical class is driven by computational demands. If correct, one would expect to find areas in the left prefrontal cortex more strongly activated for nominal than verbal morphological operations in Chinese, and none for the reverse comparison. On the other hand, if neural representation of grammatical morphemes does not simply reflect processing demand but in fact is form class specific, it is possible to find separate neural correlates for classifiers and aspect markers.

We carried out two experiments, a production task (sentence completion) in which the participants supplied either a sortal classifier or an aspect marker to complete a sentence, and a grammaticality judgment task. In the latter, grammatical violation arose from inappropriate pairing between a noun and a classifier, or from the incongruity between an aspect marker and the lexical aspect (or semantic structure) of a verb. For instance, an atelic verb (i.e. a verb without an inherent end point, such as stative or psych verbs) followed by a perfective marker, e.g. 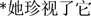 she-treasure-PERF-it, or a telic verb coupled with a continuous marker, e.g. 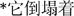 it-collapse-CONT, would result in ungrammaticality. Note that while this task could also be considered semantic judgment task as in [42], [43], [44], we prefer the term “grammaticality” since judgments in our experiment were not solely semantically based. Different from the exclusive use of object nouns in [42], [44], our stimuli included both abstract and concrete nouns in the classifier condition. Hence, congruency judgment between an abstract noun and a classifier and that between the semantic structure of a verb and the grammatical meaning of an aspect marker were not driven only by semantic features in the typical sense.

Similar to [25], [26], conjunction analyses were conducted in the production experiment to identify brain areas that were more activated for classifiers than aspect markers as well as those that were more active for verbal than nominal grammatical morphemes across concreteness conditions. The use of both concrete and abstract items and conjunction analyses across concreteness levels is an important aspect of the current design. It allowed us to identify regions that cannot be said to be mainly responsive to semantic features such as shape, function, and animacy in the case of classifiers. The classifier-specific and aspect marker-specific regions then served as regions-of-interest (ROI) to detect differential activation in grammaticality judgment to the classifier vs. aspect marker conditions. Task-independent regions specifically activated for a grammatical morpheme type were considered for their associated cognitive processes.

## Methods

### Ethics Statement

Informed written consent was obtained from all participants before the study began. The experiments were performed in accordance with the Declaration of Helsinki with ethical approval from the Institutional Review Board of the State Key Laboratory of Cognitive Neuroscience and Learning in Beijing Normal University as well as the University of Hong Kong Human Research Ethics Committee for Non-Clinical Faculties.

### Participants

Sixty-six native Mandarin speakers were recruited from Beijing Normal University to participate in the current study. Among them, 19 participants (11 female, Age mean  = 23.9, *SD* = 4.40) took part in a pilot experiment. The remaining 47 participants carried out one of the imaging experiments, with 27 (16 females, Mean age  = 20.8, *SD* = 2.14) in the grammaticality judgment experiment, and 20 (10 females, Mean age  = 21.3, *SD*  = 3.00) in the sentence completion experiment. All subjects who took part in the fMRI experiments were further required to be right-handed (assessed by the Edinburgh inventory, [45]), have normal or corrected to normal visual acuity, as well as no history of psychiatric or neurological disorders.

### Grammaticality Judgment Experiment

#### Materials and stimuli

Two aspect markers (ASPs) -- 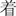 (*zhe5*, continuous ASP) and 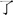 (*le5*, perfective ASP) were selected for verbs of both concrete and abstract concepts, while four sortal classifiers (CLs) were chosen with two— 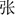 *zhang1* and 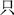 *zhi1* for concrete nouns and two -- 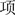 *xiang4* and 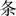 *tiao2* for abstract items. Sixty unambiguous nouns and 60 unambiguous verbs that satisfied the following criteria were selected: 1) for each word, the frequency as the target grammatical class is at least 10 times larger than that of the second most-frequently used word class; 2) for each word class, half of the items were concrete, and half were abstract; 3) nouns and verbs were balanced in frequency; 4) half of the nouns or verbs at each concreteness level were congruent with one of the CLs or ASPs but incongruent with the other of the same concreteness level, and vice versa for the other half (except for the abstract ASP condition, in which 16 verbs were congruent with the perfective maker 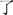 *le5* and 14 verbs with the continuous marker 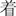 *zhe5*). Moreover, while half of the concrete verbs were transitive and the other half were intransitive, abstract verbs were all intransitive. Properties of the stimuli are given in Table 1. Note that although imageability was not balanced in the concrete level between CL and ASP sentences, a typical tendency of higher imageability of concrete nominal than vebal items, this would not affect the outcomes given our purpose and analytic method to identify areas that responded to CL and ASP sentences regardless of concreteness.

**Table 1:** Properties of materials in grammaticality judgment experiment with error rate (%) and response latency (ms) from pilot study.

Condition	Grammatical morphemes	Log frequency of noun/verb	Imaegability (SD)	No.	Example of grammatical sentence	Error (%) (SD)	RT (ms) (SD)	No.	Example of ungrammatical sentence	Error (%) (SD)	RT (ms) (SD)
CCL	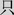 *zhi,* 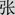 *zhang1*	1.14 (0.54)	6.6 (0.2)	30	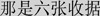 ‘Those are six CL receipts'	5.4 (8.2)	1274 (268)	20	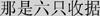 ‘Those are six CL receipts’	4.5 (4.6)	1366 (173)
CASP	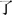 *le5*, 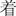 *zhe5*	1.17 (0.30)	5.0 (0.4)	30	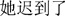 ‘She was late ASP’	3.7 (5.7)	1196 (219)	20	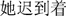 ‘She was late ASP’	11.6 (6.1)	1361 (241)
ACL	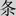 *tiao2*, 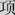 *xiang4*	1.50 (0.83)	3.5 (0.5)	30	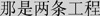 ‘Those are two CL projects’	10.2 (8.5)	1369 (206)	20	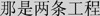 ‘Those are two CL projects’	11.3 (11.6)	1410 (187)
AASP	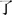 *le5*, 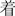 *zhe5*	1.54 (0.58)	3.5 (0.3)	30	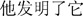 ‘He has invented ASP it’	6.0 (8.5)	1349 (340)	20	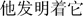 ‘He has invented ASP it’	9.2 (8.5)	1425 (182)
T-test (concrete level)		t_58_ = 0.27	t_58_ = 18.81***			t_58_ = 0.96	t_58_ = 1.2			t_38_ = 4.2***	t_38_ = 0.07
T-test (abstract level)		t_58_ = 0.23	t_58_ = 0.46			t_58_ = 1.92	t_58_ = 0.28			t_38_ = 0.65	t_38_ = 0.26

*Note*. CCL  =  concrete classifier; CASP  =  concrete aspect marker; ACL  =  abstract classifier; AASP  =  abstract aspect marker; RT  =  response latency.

*** *p<*0.001.

Simple sentence structures were used for constructing the stimuli in all conditions. This can be seen as an attempt to improve on some previous work examining morphological operations of nouns and verbs in which stimuli of different syntactic structures were employed for the noun (e.g. a noun phrase, *a boy*, *many ideas*) and verb (e.g. a verb phrase or more accurately a sentence, *I gasp*, *he sings*) conditions [11], [15], [46]. CL sentences had the structure of “this/that/these/those + is/are + noun phrase (number + CL + noun)”, with the demonstrative and number (from one to 10) randomly assigned. The ASP sentences contained “pronoun (he/she/it/they) + verb + ASP + pronoun (null for intransitive verbs), where the selection of pronouns was random across stimuli. Following the above steps, 60 grammatical CL sentences were created by inserting the nouns and corresponding CLs, and 60 grammatical ASP sentences were constructed using the verbs followed by an appropriate ASP. The same number of ungrammatical sentences, i.e. 120, was then generated by replacing the correct CL or ASP with the other one (of the same concreteness level for CL).

A pilot study was conducted with 19 participants, in order to evaluate the acceptability of the stimuli as well as the processing demand for each condition as reflected in response latency (RT). All of the grammatical sentences were accepted by more than half (i.e. 10) of the participants (see error rates in Table 1); however, some of the ungrammatical sentences were not rejected by more than 10 subjects. Therefore, only 20 out of the 30 ungrammatical sentences were selected from each condition. For the abstract CL condition, 15 ungrammatical sentences took *tiao2* and five had *xiang4* as the incongruent CL, whereas for the other three conditions, the selected CLs or ASPs were equally represented. Based on the pilot results, both the grammatical and ungrammatical sentences could be balanced on processing demand in terms of error rates and RTs between CL and ASP conditions of both concreteness levels, except that ungrammatical CL sentences with concrete nouns showed a lower error rate than concrete ASP sentences (Table 1). One point worthy of mention is that, regardless of concreteness level or sentence structure, CL sentences with six characters were significantly longer than ASP sentences, which include four to six characters. Possible effects of sentence length were addressed in data analysis.

Four additional words (two nouns and two verbs), other than experimental materials, were selected and used to construct two grammatical and two ungrammatical sentences, functioning as lead-in trials during scanning.

#### Design and procedure

An event-related design was adopted. Sentences from each condition were combined and divided into four blocks of 50 items, with grammatical and ungrammatical sentences associated with the same CL or ASP condition distributed equally across blocks. Care was further taken to ensure that grammatical and ungrammatical trials containing the same nouns or verbs were not assigned to the same block. Items in each block were arranged according to optimal event type scheduling computed by Optseq software (http://surfer.nmr.mgh.harvard.edu/optseq/), forming one experimental run with one lead-in trial added at the beginning. The order of the four experimental runs was counterbalanced across participants.

The experiment was conducted in E-prime 1.2. During each run, a blank screen would first appear and last for 10 s, allowing the participants to adjust to the scanning environment. The lead-in trial then appeared, followed by one experimental block. In each trial, a sentence stimulus would be presented in the center of the screen (visual angle: 5° ∼7° depending on the sentence length) for 4 s. Participants were instructed to judge whether the sentence was grammatical or not by pressing the corresponding buttons with their left hand, as accurately and quickly as possible. Response accuracies and latencies were recorded. The stimulus was replaced by a blank screen, the duration of which was determined by the Optseq software (min = 2 s, max = 18 s, mean = 4 s), in order to optimize the event scheduling for better partition and estimation of each event type. The next trial began after the jittered ISI (inter-stimulus interval). Throughout the run, a red dot remained in the center of the screen as the fixation point.

Each run lasted about 7 minutes, and there was a self-paced break between runs (around 2 minutes). The entire experiment, including the practice and preparation, took approximately 50 minutes.

#### MRI data acquisition, preprocessing and first-level analysis

Functional MRI scans were collected on a 3.0 Tesla Siemens scanner using a 12-channel transmit/receive gradient head coil (Beijing Normal University, China). A T2*-weighted gradient-echo planar imaging (EPI) sequence was applied to acquire the blood oxygen level-dependent (BOLD) signals (flip angle 90°, TE = 30 ms, TR = 2000 ms, in-plane resolution = 3.125*3.125, slice thickness  = 4 mm, slice gap = 0.8 mm).

Data preprocessing and analysis were performed using SPM5 (http://www.fil.ion.ucl.ac.uk/spm/software/spm5/). The first 9 TRs containing blank screen and lead-in trials were deleted from each run, before functional images were slice-time and head motion corrected for each run per subject. Subsequently, data were normalized to a standard template in Montreal Neurological Institute (MNI) space and then smoothed with an isotropic 8-mm full-width-half-maximal Gaussian kernel.

For the first-level analysis, due to excessive head movement (> 2 mm or 2°) within at least one experimental run, four participants (three females) were excluded from further analysis. The images of the other 23 participants were entered into two models that were set up for different purposes.

A. Conventional model: this model was built to investigate the processing of different grammatical morphemes. Thus, eight event types, as decided by experimental manipulations (2 grammatical morpheme types * 2 concreteness levels * 2 grammaticality levels) were considered and modeled with the canonical hemodynamic response functions for estimation. The high-pass filter was set at 273 s, calculated based on the longest time interval between trials from the same condition. After model estimation, subjectwise contrast maps were computed for each of the following four conditions against fixation, which were grammatical and ungrammatical CL sentences (GCL, UCL), as well as grammatical and ungrammatical ASP sentences (GASP, UASP).

B. Length model: The second model was constructed to evaluate the effect of sentence length in terms of number of characters as it was not balanced across experimental conditions, which might have confounded with effects of contrasts between nominal and verbal grammatical morphemes. ASP sentences with concrete verbs were combined across grammaticality and re-divided based on verb transitivity into sentences with transitive verbs (Verb_transitive_, 19 trials) and those with intransitive verbs (Verb_intransitive_, 31 trials). Sentences in the former condition contained five or six characters, which were significantly longer than those in the latter condition (four or five characters, Mean length: Verb_transitive_ = 5.2, Verb_intransitive_ = 4.3; *t*(48) = 6.9, *p*<0.001). During model specification, Verb_transitive_ and Verb_intransitive_, as well as the other six event types as in the conventional model, were fed into the GLM. The high-pass filter was adjusted to 286 s. Subjectwise contrast maps for Verb_transitive_ versus fixation, as well as Verb_intransitive_ versus fixation were produced for subsequent analysis.

#### Chronometric data analysis

Subjectwise accuracies were first computed by averaging accuracies across all items. One participant with accuracy lower than 80% was excluded from further analysis. Thus, due to excessive head movement and/or poor performance, five participants in total were removed, leaving data from 22 subjects (13 females) for further behavioral and imaging data analyses.

For behavioral analyses, RT data were trimmed if responses were incorrect, absent, or 3 *SD*s away from the individual mean. Error rates and RTs were then entered into three-way ANOVA tests with item and subject as random factors, respectively, to evaluate the main effects of concreteness, word class and grammaticality, as well as their interactions. Results were considered reliable only if both by-item and by-participants analyses were significant.

### Sentence Completion Experiment

#### Materials and stimuli

The 120 grammatical sentences in the grammaticality judgment task served as materials in this experiment (see Appendix for the entire list of sentences). The stimuli were created by masking the CL or ASP in each sentence. Four sentence stimuli (two CL and two ASP sentences) with nouns and verbs other than the experimental materials were further created, as lead-in trials in the imaging experiment.

Moreover, 50 words randomly selected from the experimental materials and 39 novel words other than the stimuli in the experiment were employed in a post-scanning memory probe test, in order to evaluate participants' attentiveness during scanning.

#### Design and procedure

An event-related design was adopted as in the grammaticality judgment task. Items from each condition were mixed and divided into two blocks of 60 trials. In each block, sentences types were balanced across the four conditions, and further matched on the number of trials between sentences with either CL or ASP within each condition. Similar to the judgment experiment, item sequence in each block was computed by Optseq, forming one experimental run with two lead-in trials added at the beginning. To minimize the order effect, two lists composed of two blocks with different stimulus sequences were generated and randomly assigned to the 20 participants, with 10 for each list. The run order was counterbalanced across the 10 subjects for the same list.

E-prime 1.2 was used to run the experiment. At the beginning of each run, a blank screen was first presented for 10 s, followed by lead-in and experimental trials. During each trial, an incomplete sentence would be shown in the center of the screen (visual angle: 5° ∼7°) for 4 s, during which participants were required to produce one CL (except the general CL ???*ge*) or ASP appropriate for the stimulus sentence covertly, in order to minimize head movement. The stimulus was then replaced by a blank screen with a jittered ISI (computed by Optseq, min = 2 s, max = 12, mean = 4 s) before the next trial began. Throughout the run, a red dot remained in the center as the fixation point. Each of the two runs lasted for 8.4 minutes, and there was a 2-minute break in between. Immediately after the experiment, the participants were asked to attend a memory probe test. Each subject had to indicate if a stimulus word had been seen in the scanner. The test was self-paced and took approximately three minutes to complete.

The entire experiment, including the practice, preparation, and probe test, took approximately 35 minutes. The participants were required to return the next day to repeat the same experiment outside the scanner with overt responses, in order to collect their responses and response latencies.

#### MRI data acquisition, preprocessing and first-level analysis

The parameter setting for scanning and the procedure of preprocessing were identical to those in the grammaticality judgment experiment, except that the initial 13 TRs containing blank screens and lead-in trials were removed from each run. Due to excessive head movement (> 2 mm or 2° in at least one run), data of three participants (one female) were discarded.

For subject-level analysis, two models with the same purposes as those in the grammaticality judgment experiment were built. In the conventional model, regressors for the four event types (CCL, ACL, CASP, AASP) were included, and contrast maps for each condition versus fixation were computed after estimation. The high-pass filter was set at 191 s. For the length model, trials with concrete verbs were divided into Verb_intransitive_ (18 trials, mean length = 4.3) and Verb_transitive_ (12 trials, mean length = 5.2), which significantly differed in sentence length, *t*(28) = 5.5, *p*<0.001. Five event types (Verb_transitive_, Verb_intransitive_, ASP, CCL, ACL) were entered into modeling, and contrast maps were computed accordingly. The high-pass filter was adjusted to 286 s for the second model.

#### Chronometric data analysis

The accuracy rate for the memory probe test was first calculated for each participant. Cut-off score was set at 65.2%, which was significantly above chance level. Two subjects (one of whom also had excessive head movement) with accuracies lower than the criterion were excluded. In the end, data of four participants were discarded due to excessive head movement or poor performance in the memory probe test. Data of the remaining 16 participants (nine females) underwent further statistical analyses.

The appropriateness of responses collected outside the scanner was judged by two raters, who were naïve to the design and aims of the current experiment. CLs or ASPs that were rated as ungrammatical by one of the two participants were regarded as errors. Response times were trimmed if a) an erroneous response was given, b) the voice key was triggered by noise, such as cough, or c) the value was 3 *SD*s away from the subjectwise mean or less than 200 ms. Both the error and RT results were analyzed with two-way ANOVAs with items and subjects as random effects, respectively, to calculate the main effects of grammatical morpheme and concreteness, as well as their interaction. Similar to the grammaticality judgment experiment, results were considered reliable only if both by-item and by-participants analyses were significant.

### Imaging Data Analysis Involving Sentence Completion and Grammaticality Judgment Tasks

Group-level analyses of imaging data were conducted across the two experiments to reveal task-independent effects. Regions more strongly activated for the CL or ASP condition were first obtained from the sentence completion experiment by a whole-brain analysis. Contrast maps of each condition versus fixation that were computed at the first-level were fed into a 2 (grammatical morpheme) *2 (concreteness) flexible design. A conjunction analysis was conducted on the CL vs. ASP contrasts between the two concreteness levels (i.e., (CCL-CASP)∩(ACL-AASP), (CASP-CCL)∩(AASP-ACL)), in order to localize regions that were differentially activated for the CL and ASP conditions for both concrete and abstract levels. Threshold was held at voxel-level *p_unc_*<0.001, with a cluster extent threshold of 60 voxels for each contrast, in order to survive a Monte-Carlo corrected clusterwise alpha level of 0.049.

Convergence analysis of regions associated with processing of different grammatical morphemes was then conducted with data from the grammaticality judgment experiment using an ROI approach. For each region specific to CL or ASP processing in the completion experiment, percentage signal change in each of the eight conditions against fixation (conventional model) was extracted and averaged across voxels. They were entered into a two-way ANOVA with grammatical morpheme and grammaticality as fixed factors and subject as the random factor. Note that a two-way, rather than a three-way, ANOVA was conducted for the reason that concreteness was not a focus of this study. It would also be more consistent with the conjunction analysis of the sentence completion task where brain regions differentially responsive to different grammatical morphemes but regardless of concreteness were identified. A three-way ANOVA with concreteness as one of the factors was in fact carried out, and none of the ROIs showed significant interaction between grammatical morpheme and concreteness. Hence, results of the two-way ANOVA are reported. Regions replicating the grammatical morpheme effects in the sentence completion task in terms of main effect or interaction were regarded as task-independent regions for further consideration.

In addition, possible confounding effects of sentence length in both experiments were estimated in CL-specific ROIs, in order to evaluate whether the observed finding was the results of higher visual processing load due to longer sentences in the CL conditions. Based on the length effect model in each experiment, percentage signal change in the conditions of intransitive verbs and transitive verbs were extracted and averaged, respectively, for each CL-specific region. T-tests were applied to compare the activation levels between the two conditions representing different lengths. Regions showing significant length effects were regarded as neural areas sensitive to visual processing demand, which might have confounded with the effects of stronger activation apparently induced by classifiers.

## Results

### Behavioral Results

For the sentence completion experiment, the two-way ANOVA analysis of error rates did not find any significant main effect or interaction effect. However, for the RT, main effects of both grammatical morpheme and concreteness were significant (grammatical morpheme: *F_1_*(1,15) = 11.2, *p*<0.01; *F_2_*(1,116) = 15.7, *p*<0.001; concreteness: *F_1_*(1,15) = 30.5, *p*<0.001; *F_2_*(1,116) = 9.8, *p*<0.01), with items of concrete concepts and ASP sentences responded to more quickly (see descriptive results in Table 2). The interaction between grammatical morpheme and concreteness was insignificant.

**Table 2:** Behavioral results in error rate (%) and response latency (ms) of grammaticality judgment and sentence completion tasks.

Experiment	Condition	Mean (%)	SD	Mean (ms)	SD
Grammaticality judgment	Grammatical CCL	4.2	6.3	1535	188
	Grammatical CASP	5.3	7.7	1456	191
	Grammatical ACL	15.2	12.6	1654	191
	Grammatical AASP	9.4	8.2	1627	196
	Ungrammatical CCL	4.8	4.5	1546	155
	Ungrammatical CASP	7.5	5.8	1566	189
	Ungrammatical ACL	9.1	6.3	1664	131
	Ungrammatical AASP	8.2	8.7	1589	142
Sentence completion	CCL	2.7	6.5	1222	212
	CASP	3.5	5.8	1117	124
	ACL	7.7	14.2	1339	187
	AASP	2.5	5.6	1196	149

*Note*. Mean and SD were calculated across item-wise values within each condition. CCL  =  concrete classifier; CASP  =  concrete aspect marker; ACL  =  abstract classifier; AASP  =  abstract aspect marker.

For the grammaticality judgment experiment, the three-way ANOVA revealed a significant main effect of concreteness on both error rates and RTs (error rates: *F_1_*(1,21) = 34.3, *p*<0.001; *F_2_*(1,192) = 18.3, *p*<0.001; RT: *F_1_*(1,21) = 47.5, *p*<0.001; *F_2_*(1,192) = 17.4, *p*<0.001) with concrete items easier and quicker to respond to, while main effects of grammatical morpheme and grammaticality were not significant in either error rates or RT (Table 2). The interaction between concreteness and grammaticality for error rates (*F_1_*(1,21) = 9.3, *p*<0.01; *F_2_*(1,192) = 4.6, *p*<0.01) was significant, with higher error rates for ungrammatical trials at the concrete level but a reversed tendency for trials with abstract concepts. However, post-hoc analyses did not reveal any significant simple effect (all *p*>0.1). The other two-way interactions and the three-way interaction were not statistically reliable.

### Imaging Results

Conjunction analyses of CL vs. ASP contrasts between the two concreteness levels in the sentence completion task revealed that the left posterior middle temporal gyrus (adjacent to the superior temporal gyrus) was activated more strongly for the ASP sentences than the CL sentences for both concrete and abstract levels, whereas regions showing greater activation for the CL conditions of both concreteness levels included bilateral calcarine and lingual gyri (area in the left hemisphere extended into posterior fusiform gyrus), bilateral orbital inferior frontal gyri and insula cortex (BA47, right BA47 (rBA47)), as well as the left supplementary motor area and superior medial frontal gyrus (LSMA&SMedFG). In addition, the dorsal aspect of left triangular and opercular inferior frontal gyri (LIFG, BA44), with a smaller cluster size (k = 56, corresponding to cluster-level *p* = 0.06) was also activated more strongly for CL trials and therefore included for further consideration (see detailed information on each cluster in Table 3).

**Table 3:** Results of whole-brain analysis from sentence completion experiment.

Contrasts for conjunction	Activated region	Cluster size	x	y	z^a^	T	p
(CCL-CASP) ∩ (ACL-AASP)	Left calcarine, lingual and posterior fusiform gyri	412***	−15	−93	−9	5.72	< 0.001
	Right calcarine and lingual gyri	186***	9	−87	0	5.99	< 0.001
	Left triangular and opercular inferior frontal gyri (dorsal part, BA44)	56^b^	−39	12	30	4.92	< 0.001
	Left orbital inferior frontal gyrus and insula (BA47)	225***	−33	30	−15	8.6	< 0.001
	Right orbital inferior frontal gyrus and insula (rBA47)	82*	27	27	−9	5.15	< 0.001
	Left supplementary motor area and superior medial frontal gyrus	213***	−6	27	45	4.43	< 0.001
(CASP-CCL) ∩ (AASP-ACL)	Left posterior middle temporal (adjacent to the superior gyrus)	60*	−57	−48	12	4.53	< 0.001

*Note.* For the whole brain analyses, unless specified otherwise, significant threshold was held at *p_vox_*<0.001, k≥60, corresponding to corrected cluster-level *p*<0.05. CCL  =  concrete classifier; CASP  =  concrete aspect marker; ACL  =  abstract classifier; AASP  =  abstract aspect marker.

a Peak coordinates are reported in the MNI system.

b Due to a relatively smaller cluster size, BA44 only showed a marginally significant effect of grammatical morpheme (*p_cor_* = 0.06).

* *p_cor_*<0.05, ** *p_cor_*<0.01, *** *p_cor_*<0.001.

ROI analyses of the length effect revealed two CL specific clusters – (i) left calcarine, lingual and posterior fusiform gyri, as well as (ii) right calcarine and lingual gyri -- which showed significantly greater activation for sentences with more characters (Verb_transitive_) than those with fewer characters (Verb_intransitive_) in both experiments (see Table 4). Since the confounding effects of sentence length could not be separated from those of grammatical morpheme contrasts in the current study, these two regions would not be considered further for the sake of parsimony.

**Table 4:** Results of two-way ANOVAs from grammaticality judgment and of sentence length.

Regions from conjunction analyses of sentence completion	Grammatical morpheme effect	Grammaticality effect (Ungrammatical> Grammatical)	Interaction	Length effect: Judgment task (Long > Short)	Length effect: Completion task (Long > Short)
**CL-specific regions**	(CL > ASP)				
Left calcarine, lingual and posterior fusiform gyri	***		*	***	***
Right calcarine and lingual gyri	***			***	**
BA44				*	
BA47		*	**		
rBA47		***	*		
Left supplementary motor area and superior medial frontal gyrus		*	***		
**ASP-specific regions**	(ASP > CL)				
Left posterior middle temporal	***	**		–	–

*Note.* CL  =  classifier; ASP  =  aspect marker. *p<0.05, **p<0.01, ***p<0.001.

For the remaining four CL specific regions and one ASP specific area, results of the ROI analyses using two-way ANOVAs (grammatical morpheme x grammaticality) of the grammaticality judgment task revealed a significant main effect of grammatical morpheme only in left posterior middle temporal gyrus, with larger signal changes induced by the ASP condition. This pattern was consistent with the results in the sentence completion task. The main effect of grammaticality was significant in bilateral BA47, LSMA&SMedFG, as well as left posterior middle temporal gyrus with ungrammatical sentences inducing stronger responses (Table 4).

**Figure 1:**
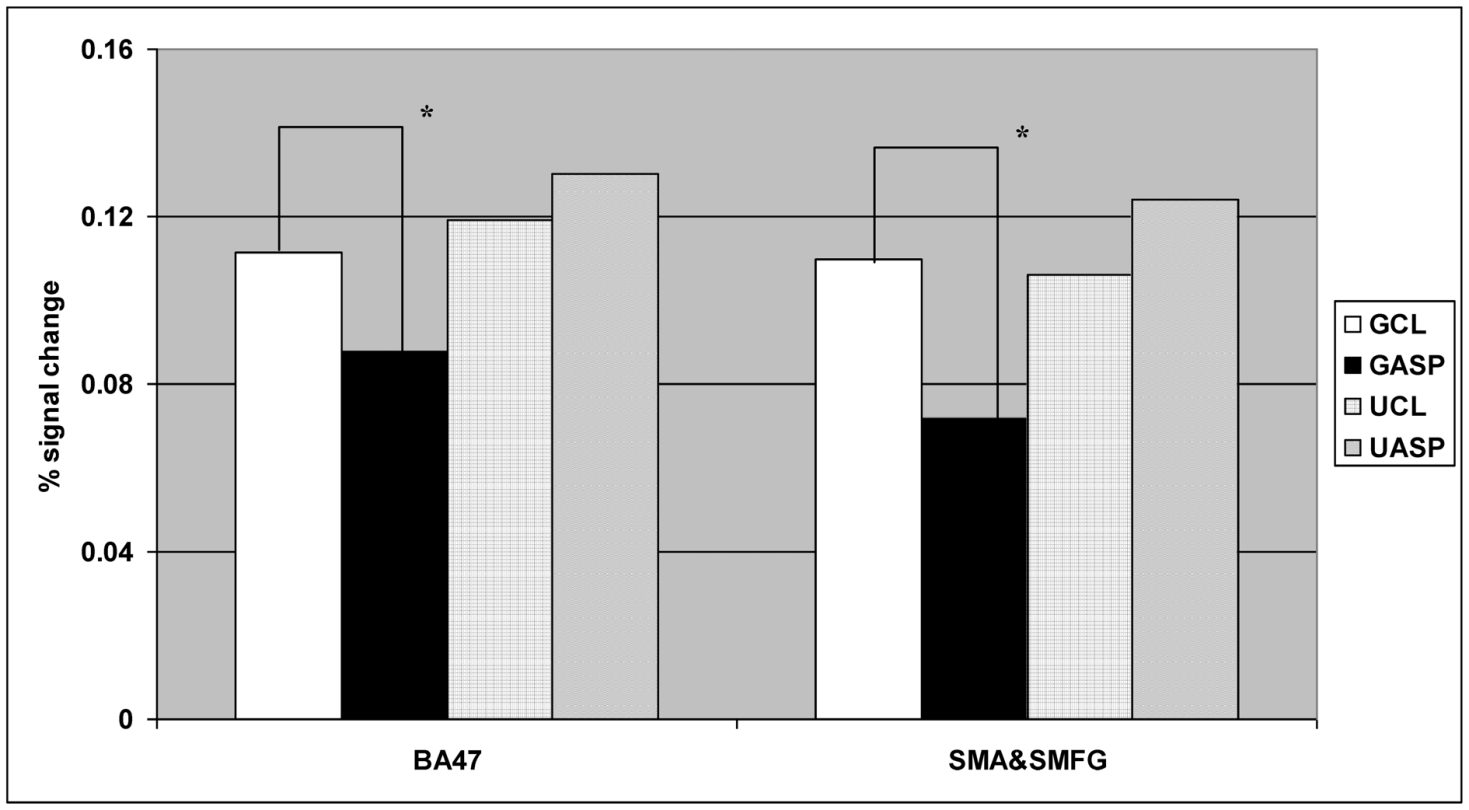
Interaction effects between grammatical morpheme type and grammaticality in left BA47 and SMA&SMedFG. *Note.* GCL  =  grammatical classifier; GASP  =  grammatical aspect marker; UCL  =  ungrammatical classifier; UASP  =  ungrammatical aspect marker; BA47 =  left Brodmann area 47; LSMA&SMFG  =  left supplementary motor area and superior medial frontal gyrus. *p<0.05.

A significant interaction effect between grammatical morpheme and grammaticality was also observed in BA47, rBA47, and LSMA&SMedFG. Among them, interaction effects in two regions -- BA47 and LSMA&SMedFG – were caused by higher activation for CL sentences than ASP sentences in grammatical trials only (Figure 1). This pattern was consistent with the results of the sentence completion task. Post-hoc analyses contrasting grammatical CL with ASP sentences found significant differences in both regions (BA47: t(21) = 2.42, p<0.05; LSMA&SMedFG: t(21) = 2.50, p<0.05). For rBA47, the interaction exhibited a pattern of lower activation for ungrammatical CL sentences than ungrammatical ASP sentences with a reversed effect between CL and ASP trials for the grammatical condition. However, post-hoc analyses did not find significant simple effects between CL and ASP sentences in either grammaticality condition (grammatical trials: *p*>0.1; ungrammatical trials: *p*≥0.07).

**Figure 2:**
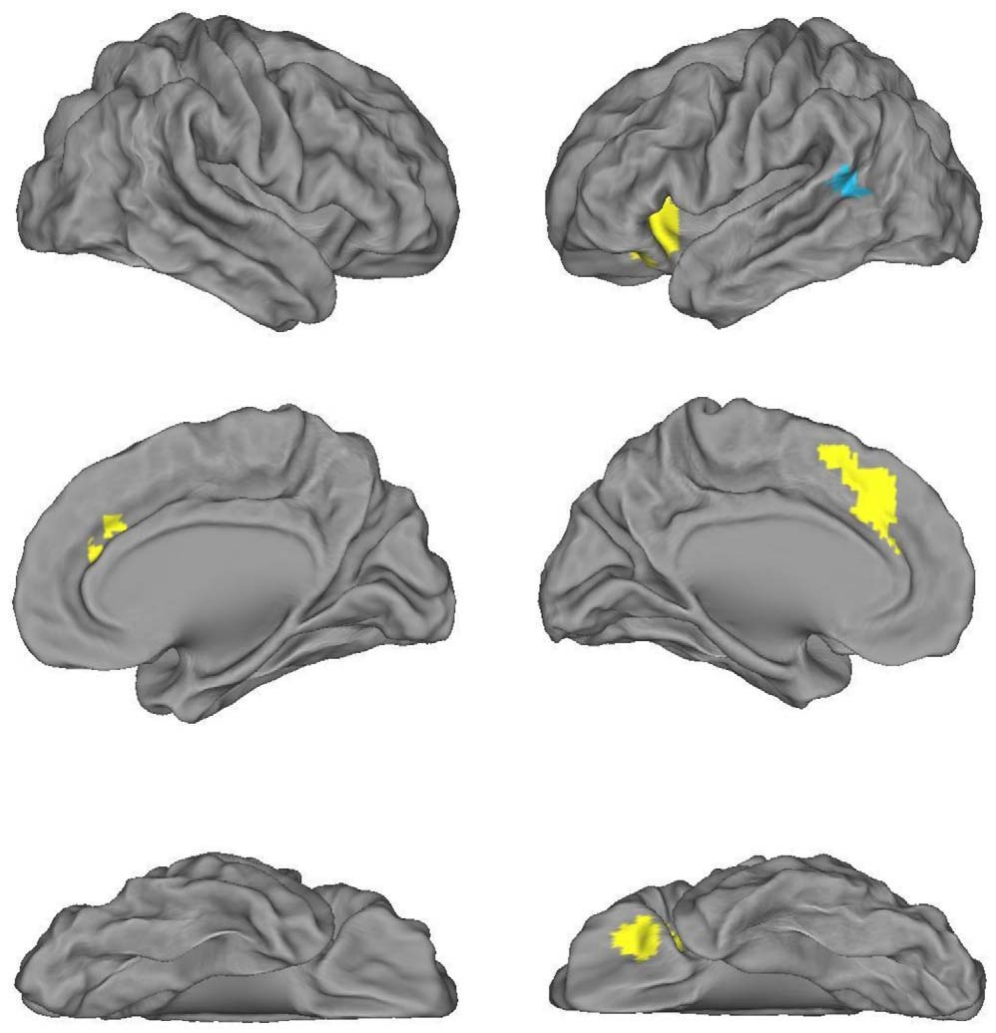
Task-independent regions of grammatical morpheme processing associated with Chinese nouns and verbs. *Note*. Classifier-specific regions are drawn in yellow and aspect marker-specific region in blue.

In summary, with respect to processing nominal classifiers and verbal aspect markers, convergence analyses have shown task-independent regions for greater response to classifiers in BA47 and LSMA&SMedFG, and to ASP stimuli in the left posterior middle temporal gyrus, as illustrated in Figure 2.

## Discussion

The neural bases underlying processing of Chinese classifiers and aspect markers were investigated through one expressive task – sentence completion, and one receptive task—grammaticality judgment, to look for converging evidence for the processing of Chinese grammatical morphemes associated with nouns and verbs. From the production task, we identified a number of regions that were more strongly activated during classifier selection and those that were more responsive to aspect marker selection, regardless of the concreteness of the relevant noun and verb. Signal changes in these regions from the judgment task were then extracted to assess effects of grammatical morphemes and grammaticality, and their interaction. Task-independent regions showing greater activation associated with classifier processing included left BA47 and SMA&SMedFG, whereas stronger response to aspect marker processing was found in the left posterior middle temporal gyrus. We consider below each of these regions in terms of its possible role in processing nominal and verbal grammatical morphemes in Chinese given previous claims that have been made about its function(s).

As discussed in the Introduction, two major functional roles relevant to the present investigation have been associated with the left ventral prefrontal cortex – domain general executive functions and language-specific functions. The domain general account contends that the prefrontal cortex supervises or coordinates with other parts of the brain to perform various cognitive activities. It functions as a control hub in extracting/gathering information from a large variety of sources and projecting back to these systems to guide some voluntary action/decision [47], or choosing an appropriate response according to the task at hand from amidst possible candidates [21]. It has also been linked to working memory, temporarily holding non-integrated pieces of information before processing [48]. As such, the activation in the prefrontal cortex is supposed to be sensitive to task difficulty, as higher task demand corresponds to heavier load exerting on computational and/or working memory resources in any task. Hence, its involvement in grammatical morpheme processing might simply reflect a difference in paradigmatic complexity between morphosyntactic operations of nouns and verbs [18], [49], [50]. Researchers have further explained the engagement of the prefrontal cortex in terms of functional or structural connections to other more domain specific regions [48], [51], e.g. LIFG – left temporal pole network for semantic processing, LIFG-left posterior MTG for syntactic processing [52], [53].

Alternatively, it has been argued that specific region(s) in the left prefrontal cortex are dedicated to linguistic processing. Moreover, functional fractionation within this region has been proposed – BA47 for semantic processing (e.g., [54], for Chinese [55], Broca's area for syntactic processing (e.g., [56], for Chinese [57]), semantic processing (e.g. [42], [44], [57] for Chinese), and morphosyntactic processing (e.g., [58]). Some studies tried to demonstrate the specific role of these areas in linguistic processing regardless of influence of working memory or selection demand by balancing computational loads (e.g., [59] for semantic processing, and [11] for morphosyntactic processing), or adding orthogonal/independent manipulation of task difficulty (e.g., [60] for semantic processing, and [61]; [62] for syntactic processing, but see [63] for alternative interpretations of [62]).

Two left prefrontal areas, BA47 and LSMA&SMedFG, were found to respond more strongly to classifier than aspect marker processing in this study. While one may interpret these areas as underlying morphological processes of nouns, it is also reasonable to propose that participants may need more computational resources, perhaps in terms of working memory or selection demand, to process CL sentences as there are more activated morphemes in the case of CL than ASP operations. This interpretation is consistent with the significantly longer RT in the classifier than ASP condition in the sentence completion task (Table 2). We are aware of previous claims from a series of Chinese studies that activation in the left inferior frontal cortex basically supports semantic processing, as the language lacks inflectional morphology, morphosyntactic processes, and purely syntactic violations [42]. However, it is important to note that all the evidence comes from tasks involving semantic judgment at the sentence level [42], [44], [57] or the lexical level [55], [64], [65], [66], [67], [68], [69]. While making judgment about semantic acceptibility or relatedness clearly involves semantic processes, processing semantic incongruency or making relatedness decision about two items may also be more resource demanding, which may or may not be reflected in response latency. Our previous finding from a semantic relatedness judgment task also identified a marginally significant cluster in BA44 related to word class effects [25], but the region was no longer significant when we looked for convergence between the judgment task and a semantic associate production task [26]. Most relevant to our consideration of the functional role of BA47is a study that employed reversible two-character Chinese words, e.g. ?? ‘to lead’ and?? ‘a necktie’, and concluded that executive control processes of semantic retrieval modulated activities in that area [55]. Interestingly, that study also found the Broca's area more responsive to the ‘low conflict’ compared with neutral condition. Given these findings in Chinese, it would be more parsimonious to attribute the stronger response for CL stimuli in BA47 and LSMA&SMedFG to higher processing demand, resulting from activation of morphemes of a larger CL inventory. One noteworthy finding in the current study was the significant interaction between grammaticality and grammatical morpheme type but without significant simple effects in rBA47. The pattern of signal changes in this region was similar to those of BA47 and LSMA&SMedFG, except that the difference between CL and ASP sentences was not statistically reliable for the grammatical trials. While this may be taken as left lateralized language functions in the prefrontal cortex, the role of rBA47 in linguistic processing deserves further investigation.

Our explanation for greater activation of CL processing in the left prefrontal cortex is analogous to the account of higher paradigmatic complexity or greater processing demand in European languages [17], [18], [50]. However, it remains unclear whether there is subdivision of functional roles associated with complexity or competition within LIFG and how they are spatially represented. BA45/47 was suggested to be involved in domain general executive control in decision making and response selection among semantic competitors (e.g. [20], [21]). BA44 was found to reflect processing demand in studies of European languages which explicitly contrasted different levels of complexity of inflectional operations [17], [18], [70], [71], but the same area was argued to underlie phonological competition (e.g. [19]). Moreover, LSMA&SMedFG was also associated with “self-initiated, self-guided retrieval of semantic information” p. 6 in [72].

Our finding of the left posterior middle temporal gyrus activated specifically for ASP sentences could be interpreted in terms of neural substrates underlying verb semantic processing, since the aspect marker indicates a temporal view of the event denoted by a verb in progressive, habitual, completion, momentary, etc. Selecting or determining whether an aspect marker is appropriate for a verb is to a large extent based on the grammatical meaning of the aspect marker and the lexical aspect of the verb. The proposed interpretation is not only consistent with our previous findings from a semantic relatedness judgment task and a semantic associate generation task with task-independent activation for Chinese verbs regardless of concreteness in the left posterior superior and middle temporal gyri [25], [26], but also compatible with studies contrasting semantic processing of different word classes showing verb-specific activation in lateral posterior temporal gyri (e.g., [41], [73], [74] and [75] for activation in bilateral posterior temporal cortex).

The observation of sensitivity of the left posterior middle temporal gyrus to the grammaticality manipulation echoes previous findings showing its involvement in semantic/syntactic integration during sentence processing. The area was more activated when processing syntactically complex than simple sentences (e.g., [76], [77]), as well as semantically and/or syntactically anomalous than normal sentences (e.g., [78], [79]). Neuropsychological studies have also shown that lesions in this region would lead to disruption in grammaticality judgment performance of English speakers with aphasia (posterior temporal areas in [80]), and of sentence comprehension possibly due to inability to integrate sentential components to achieve one cohesive message [52]. Such a combinatory account may explain the grammaticality effect as it is expected that ungrammatical sentences would require more effort to derive an interpretation due to semantic/syntactic incongruity in the sentence.

Finally, although the main findings of this study have come from brain imaging data, one aspect of the behavioral results is worth mentioning for future studies involving processing of nominal classifiers in Chinese and using abstract and concrete nouns. We reported earlier a significant two-way interaction in error rate, albeit insignificant simple effects, between grammatical morpheme and concreteness in the grammaticality judgment task with higher error rates for ungrammatical trials at the concrete level but a reversed pattern for trials with abstract content words. An examination of Table 2 suggests that the higher error rate of abstract grammatical trials than ungrammatical trials might be driven by the particularly high error rate of grammatical sentences containing abstract nouns. We propose that this may be related to the greater flexibility of use of classifier for abstract concepts. That is, compared with most concrete nouns with specific classifiers, there is relatively lower agreement on the most appropriate classifier for an abstract noun.

In conclusion, through contrasting the processing of classifiers and aspect markers representing, respectively, nominal and verbal morphological operations in receptive and expressive tasks, we have found converging evidence for brain regions differentially responsive to one type of stimuli over the other, and vice versa. We have attributed the activation in the left prefrontal cortex to greater paradigmatic complexity of classifiers than aspect markers, which may reflect domain general computational loads, consistent with views from studies of European languages [18], [19], [20], [21], [70], and the left posterior temporal gyrus to more demanding verb semantic processing stemming from congruency between aspect markers and semantic structure of verbs. Our results have contributed for the first time to cross-linguistic study of neural representation of grammatical morpheme processing from an analytic and a classifier language.

## Supporting Information

Appendix S1 **Sentence stimuli containing nominal classifiers or verbal aspect markers in sentence completion and grammaticality judgment tasks.**
(DOCX)
