# Supplementary material for: An fMRI Study of Grammatical Morpheme Processing Associated with Nouns and Verbs in Chinese
Source: PLoS One. 2013 Oct 11;8(10):e74952. doi: 10.1371/journal.pone.0074952 (PMC3795710; doi:10.1371/journal.pone.0074952)
Supplement: Appendix S1 — Sentence stimuli containing nominal classifiers or verbal aspect markers in sentence completion and grammaticality judgment tasks. (DOCX) [file pone.0074952.s001.docx]

|  | Classifier Condition |  |  | Aspect marker Condition |  |
| --- | --- | --- | --- | --- | --- |
| Noun | Meaning | Sentence | Verb | Meaning | Sentence |
| Concrete | |  |  |  |  |
| 蚂蚁 | Ant | 那是两只蚂蚁。 | 点燃 | ignite | 她点燃了它。 |
| 蜻蜓 | dragonfly | 这是三只蜻蜓。 | 外出 | go out | 他外出了。 |
| 苍蝇 | Fly | 这是七只苍蝇。 | 打开 | open | 她打开了它。 |
| 眼睛 | Eye | 这是九只眼睛。 | 倒塌 | collapse | 它倒塌了。 |
| 饭碗 | bowl | 这是五只饭碗。 | 丢掉 | throw away | 他丢掉了它。 |
| 燕子 | swallow | 那是八只燕子。 | 醒来 | wake up | 她醒来了。 |
| 熊猫 | panda | 这是十只熊猫。 | 上来 | come up | 他上来了。 |
| 袜子 | sock | 那是七只袜子。 | 迟到 | be late | 她迟到了。 |
| 耳朵 | Ear | 那是一只耳朵。 | 去世 | pass away | 她去世了。 |
| 天鹅 | swan | 这是五只天鹅。 | 逮捕 | arrest | 他逮捕了他。 |
| 镯子 | bracelet | 那是四只镯子。 | 站住 | stand | 她站住了。 |
| 拳头 | Fist | 那是八只拳头。 | 遇见 | meet | 她遇见了他。 |
| 杜鹃 | cuckoo | 那是两只杜鹃。 | 找到 | find | 她找到了他。 |
| 狐狸 | Fox | 那是十只狐狸。 | 坐下 | sit down | 他坐下了。 |
| 手表 | watch | 那是三只手表。 | 熄灭 | extinguish | 它熄灭了。 |
| 照片 | photo | 那是五张照片。 | 注视 | stare | 他们注视着它。 |
| 海报 | poster | 这是八张海报。 | 凝视 | gaze | 她凝视着它。 |
| 试卷 | examination paper | 那是五张试卷。 | 屹立 | stand | 它屹立着。 |
| 证件 | Identity document | 这是三张证件。 | 守候 | wait | 她守候着他。 |
| 名片 | name card | 那是两张名片。 | 吆喝 | call out | 他吆喝着。 |
| 钞票 | paper money | 这是七张钞票。 | 游玩 | play | 他们游玩着。 |
| 报纸 | newspaper | 这是四张报纸。 | 端坐 | sit straight | 他们端坐着。 |
| 请柬 | invitation | 那是一张请柬。 | 等候 | wait | 他等候着她。 |
| 表格 | form | 那是两张表格。 | 奔波 | rush about | 他们奔波着。 |
| 收据 | receipt | 那是六张收据。 | 毗邻 | be adjacent to | 它毗邻着它们。 |
| 门票 | ticket | 这是七张门票。 | 行走 | walk | 他行走着。 |
| 墙纸 | wall paper | 这是两张墙纸。 | 穿行 | pass through | 他们穿行着。 |
| 图画 | drawing | 这是十张图画。 | 围绕 | surround | 他围绕着她。 |
| 地图 | map | 那是五张地图。 | 盘旋 | hover | 它盘旋着。 |
| 布告 | bulletin | 那是四张布告。 | 嬉戏 | play | 他们嬉戏着。 |
|  | |  |  |  |  |
| Abstract | |  |  |  |  |
| 途径 | approach | 那是三条途径。 | 出卖 | betray | 他出卖了她。 |
| 思路 | thought | 这是两条思路。 | 理解 | understand | 他理解了她。 |
| 线索 | clue | 这是三条线索。 | 突破 | break through | 他突破了它。 |
| 典故 | story | 这是两条典故。 | 疏忽 | neglect | 他们疏忽了她。 |
| 路线 | route | 那是九条路线。 | 得罪 | offend | 他得罪了她。 |
| 俗语 | saying | 这是四条俗语。 | 培养 | cultivate | 他培养了她。 |
| 成语 | idiom | 那是一条成语。 | 侦破 | detect | 他侦破了它。 |
| 要领 | gist | 那是一条要领。 | 抛弃 | abandon | 她抛弃了它。 |
| 原理 | principle | 这是一条原理。 | 解放 | liberate | 他解放了他们。 |
| 语句 | phrase | 这是八条语句。 | 取消 | cancel | 她取消了它。 |
| 热线 | hotline | 那是一条热线。 | 原谅 | forgive | 她原谅了他。 |
| 退路 | a way out | 那是六条退路。 | 排除 | exclude | 他排除了它。 |
| 谜语 | riddle | 这是一条谜语。 | 批准 | permit | 她批准了它。 |
| 流言 | rumor | 这是六条流言。 | 招收 | recruit | 她招收了它。 |
| 生命 | Life | 那是两条生命。 | 发明 | invent | 他发明了它。 |
| 业务 | business | 那是六项业务。 | 设立 | set up | 他设立了它。 |
| 仪式 | ceremony | 这是七项仪式。 | 保护 | protect | 他保护着她。 |
| 费用 | expense | 那是八项费用。 | 激励 | encourage | 他们激励着他。 |
| 壮举 | feat | 这是三项壮举。 | 支持 | support | 她支持着他。 |
| 联赛 | league match | 这是八项联赛。 | 痛恨 | hate | 他痛恨着她。 |
| 任务 | duty | 这是五项任务。 | 向往 | yearn | 他向往着它。 |
| 典礼 | ceremony | 这是五项典礼。 | 指望 | count on | 他们指望着他。 |
| 经费 | fund | 那是三项经费。 | 渴求 | crave | 他们渴望着它。 |
| 科研 | research | 那是两项科研。 | 憧憬 | look forward to | 他们憧憬着它。 |
| 资金 | fund | 那是一项资金。 | 热衷 | be keen | 他热衷着它。 |
| 冠军 | champion | 这是两项冠军。 | 期待 | anticipate | 她期待着它。 |
| 事业 | career | 那是四项事业。 | 珍视 | cherish | 她珍视着它。 |
| 课题 | topic | 这是三项课题。 | 眷恋 | emotionally attached to | 他眷恋着她。 |
| 工程 | project | 那是两项工程。 | 酷爱 | love | 他们酷爱着它。 |
| 基金 | mutual fund | 这是四项基金。 | 敬爱 | respect | 他敬爱着她。 |

*Note*. The underlined characters are target classifiers and aspect markers, i.e. , 只and 张in sentences containing concrete nouns, 条and 项in sentences with abstract nouns, and 了and 着 for concrete and abstract verbs.
